# Intraductal digestive enzyme accumulation drives lethal hemorrhagic necrotizing pancreatitis and enables therapeutic intervention

**DOI:** 10.21203/rs.3.rs-7861443/v1

**Published:** 2026-03-06

**Authors:** Baoan Ji, Jiale Wang, Yang Liu, Lingxiang Wang, bin wang, Jianhua Wan, Gordon He, Yasmin Jahan-mihan, Audrey Li, Yuxi Wang, Donfeng Feng, Jie Hu, Justin Clark, Muhammad Faheem, Oliver Wang, Zhu Zhu, Guangping Tu, Xiao Yu, Richard Waldron, Aurelia Lugea, Stephen Pandol, Brandy Edenfield, lizhi zhang, Ying Wang, Yan Bi

**Affiliations:** Mayo Clinic; Mayo Clinic; Mayo Clinic; Mayo Clinic; Mayo Clinic; Mayo Clinic; Mayo Clinic; Mayo Clinic; Mayo Clinic; Mayo Clinic; Mayo Clinic; Mayo Clinic; Mayo Clinic; Mayo Clinic; Mayo Clinic; The Third Xiangya Hospital, Central South University; Third Xiangya Hospital, Central South University; Cedars-Sinai Medical Center; Mayo Clinic; Mayo Clinic

## Abstract

**Background:**

Hemorrhagic necrotizing pancreatitis (HNP) is a highly lethal form of pancreatitis that lacks mechanism-based therapy. Intra-acinar enzyme activation is fundamental to pancreatitis initiation, yet it does not explain why only a subset of episodes progress to catastrophic hemorrhagic necrosis.

**Objective:**

To identify determinants of progression to lethal HNP and test a mechanism-based preventive intervention.

**Design:**

Using humanized trypsinogen mouse models and secretagogue regimens, we compared high-dose cerulein with low/moderate-dose cerulein and bombesin. We quantified pancreatic protease activity, performed blinded histopathologic scoring, assessed hemorrhage/vascular disruption, and evaluated enzyme localization and ductal injury by PRSS1 immunostaining. De-identified human pancreatic histology from HNP were examined. We tested a repurposed strategy using clinically approved agents for other indications: secretin to stimulate ductal fluid secretion and isosorbide mononitrate (ISMN) to promote ductal outflow via sphincter of Oddi relaxation.

**Results:**

High-dose cerulein induced robust intrapancreatic protease activation but predominantly caused edematous pancreatitis. Paradoxically, bombesin and low/moderate-dose cerulein elicited lower total pancreatic protease activity yet produced severe, frequently lethal HNP with vascular disruption and lobular ischemic necrosis. HNP was associated with intraductal enzyme accumulation and ductal injury. Secretin or ISMN alone showed limited protection, whereas combined secretin+ISMN prevented hemorrhagic necrotizing injury.

**Conclusion:**

Pancreatitis severity is determined not only by the magnitude of enzyme activation but also by where enzyme activity is exerted, implicating intraductal enzyme accumulation as a mechanistic determinant and therapeutic target in HNP. Coordinated enhancement of ductal secretion and outflow with clinically approved agents prevents HNP in vivo, supporting a translatable prevention strategy.

## INTRODUCTION

Acute pancreatitis (AP) is a common, potentially life-threatening inflammatory disorder and a leading cause of gastrointestinal hospitalization worldwide. In the United States, AP accounts for roughly 275,000 hospital admissions annually and more than $2.5 billion in direct inpatient costs^1, 2, 3^. Although most cases are mild and self-limiting, approximately 20% of patients develop severe disease with pancreatic necrosis, hemorrhage, and/or multiorgan failure^1, 2, 3^. Hemorrhagic necrotizing pancreatitis is among the most lethal presentations, with reported mortality approaching 30–40% despite modern intensive care. Yet management remains largely supportive, and no disease-modifying therapy has reliably prevented progression from early injury to hemorrhagic necrosis^4, 5^. This persistent therapeutic gap reflects an incomplete understanding of the mechanisms that determine pancreatitis subtype and severity.

The prevailing paradigm centers on inappropriate intra-acinar activation of digestive enzymes, particularly trypsinogen, as the initiating event^6, 7^. Decades of experimental work have reinforced this framework and shaped commonly used animal models, most notably supraphysiologic cerulean stimulation^8^. However, cerulein hyperstimulation predominantly produces edematous pancreatitis and does not recapitulate the hemorrhagic necrotizing phenotype observed in a subset of patients^8, 9^. Moreover, the intra-acinar activation paradigm does not explain why only a fraction of patients progress to vascular destruction, hemorrhage, and lobular necrosis. Early-stage human pancreatic tissue is rarely available, and by the time surgical or autopsy samples are obtained, widespread necrosis obscures initiating events^10, 11, 12^. As a result, the determinants that drive progression to hemorrhagic necrotizing pancreatitis remain poorly defined, hindering targeted therapeutic development.

In a recent effort to model human hereditary pancreatitis, we generated humanized PRSS1^R122H^-PRSS2 mice using a bacterial artificial chromosome together with a control line expressing wild-type human PRSS1 and PRSS2 (PRSS1-PRSS2). Homozygous PRSS1^R122H^-PRSS2 mice develop spontaneous pancreatitis by 21 days of age, whereas heterozygous mice lack spontaneous disease but are markedly sensitized to secretagogue-induced injury. Notably, a single low dose of cerulein (2.5 μg/kg) is sufficient to trigger pancreatitis in heterozygous PRSS1^R122H^-PRSS2 mice compared with PRSS1-PRSS2 controls^13^. Cerulein acts through the cholecystokinin (CCK) receptor on acinar cells to induce trypsinogen activation and proinflammatory signaling^14, 15, 16^. Because bombesin receptors are functional in both human and murine acinar cells^17, 18^, yet bombesin elicits measurable trypsinogen activation without causing pancreatitis in wild-type rodents^19, 20^, we asked whether PRSS1^R122H^-PRSS2 mice are similarly sensitized to bombesin and whether bombesin elicits a distinct pancreatitis phenotype compared with cerulein.

In this study, we found that bombesin elicited only modest trypsin activation yet unexpectedly induced severe, lethal hemorrhagic necrotizing pancreatitis (HNP). Interestingly, low-dose cerulein paradoxically resulted in severe HNP, whereas high-dose cerulein produced only edematous pancreatitis. Further analyses indicated that pancreatitis severity is determined not solely by the magnitude of enzyme activation, but by the compartment in which enzymatic activity acts on. Specifically, intraductal accumulation of digestive enzymes triggers lethal HNP. Guided by this duct-centered mechanism, we showed that pharmacologic reduction of intraductal enzyme burden with two repurposed, FDA-approved agents prevents progression to HNP in vivo, providing a readily translatable strategy to avert this devastating complication.

## MATERIALS AND METHODS

### Animals

Generation and characterization of PRSS1-PRSS2 and PRSS1^R122H^-PRSS2 transgenic mice have been described previously^13^. C57BL/6J mice were purchased from Jackson Laboratory or bred in-house from the same foundation stock. Sample sizes are detailed in the figure legends. Unless otherwise specified, data represent pooled results from three independent experiments, each including 5-10 mice per pancreatitis group and 2-5 mice per saline control group. All mice were 10-20 weeks old and sex- and age-matched across experimental groups. All animal procedures were approved by the Institutional Animal Care and Use Committee (IACUC) of the Mayo Clinic.

### Human tissue specimens

Human pancreatic tissue samples with histologically confirmed hemorrhagic-necrotizing pancreatitis were obtained under an Institutional Review Board (IRB)–approved protocol at Mayo Clinic. All procedures complied with institutional and national ethical guidelines. Formalin-fixed, paraffin-embedded (FFPE) sections were stained with hematoxylin and eosin (H&E) using standard histopathological methods and independently reviewed by board-certified pathologists. Patient identifiers were removed prior to analysis. Representative images were selected from cases showing extensive parenchymal necrosis and intralobular hemorrhage for comparison with experimental mouse models.

### Secretagogue-induced pancreatitis models

Cerulein (CCKS-001A; CPC Scientific) and bombesin (Bomb-001A; CPC Scientific) were prepared in sterile 0.9% saline at 200 μg/mL. To induce acute or chronic pancreatitis, mice received intraperitoneal (i.p.) injections of cerulein or bombesin (100 μg/kg) at 1-hour intervals for a total of eight doses. For cerulein titration studies, a single i.p. injection was administered at the indicated dose. Mice were euthanized by CO_2_ inhalation at prespecified time points. In acute models, tissues were harvested 24 h after the final injection; for progression and chronic models, mice were collected at defined intervals following the first injection. The pancreas and other organs were processed for histological and biochemical analyses. All experimental mice were maintained on a C57BL/6J background.

### Experimental therapeutic studies

Acute hemorrhagic–necrotizing pancreatitis was induced by intraperitoneal (i.p.) administration of bombesin (100 μg/kg) given as three injections at 1-h intervals. Following the final bombesin injection, mice were randomized to one of four treatment groups: (i) NS (sterile 0.9% saline, i.p.); (ii) secretin (Sct; 0.2 μg/kg; CPC Scientific, SECT-002A; i.p.); (iii) isosorbide mononitrate (ISMN; 20 mg/kg; TargetMol; i.p.); or (iv) secretin plus ISMN (Sct 0.2 μg/kg + ISMN 20 mg/kg; i.p.). Treatments were administered as a single i.p. injection at 30 min after the final bombesin dose. Group allocation was performed using random assignment; investigators were blinded to treatment during outcome assessment where feasible. All other materials and methods are described in detail in the Supplementary Material.

## RESULTS

### Bombesin induces lethal hemorrhagic necrotizing pancreatitis in humanized PRSS1 ^R122H^-PRSS2 mice.

**Figure 1:**
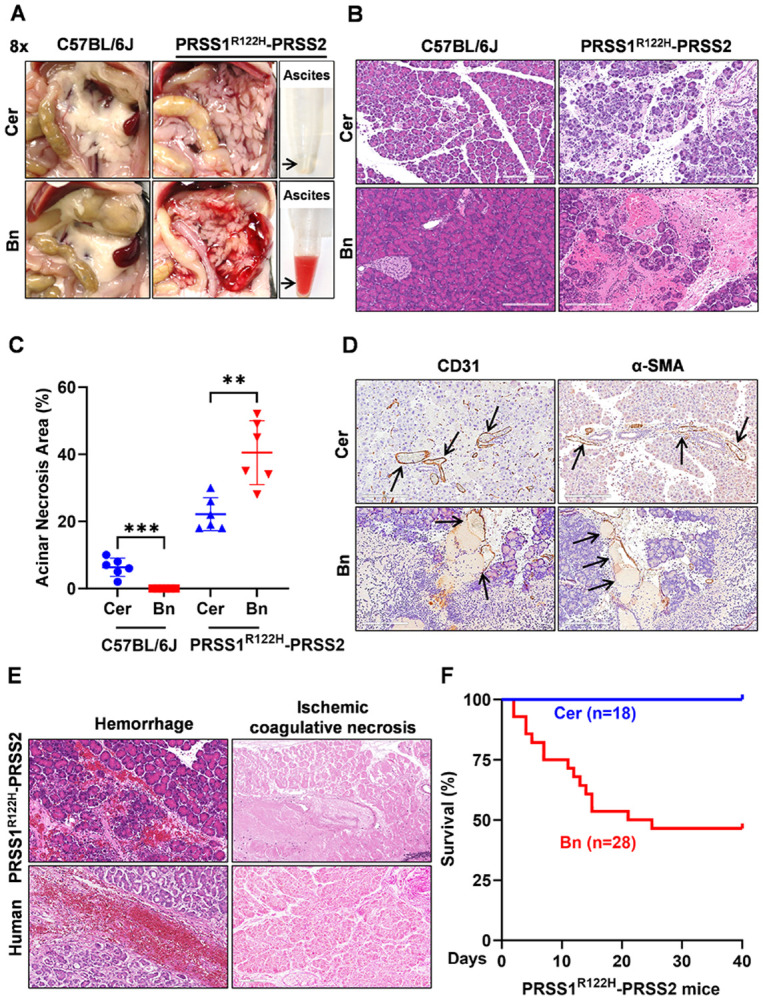
Kaplan-Meier survival analysis demonstrates ~50% mortality in bombesin-treated mice and no mortality in high-dose cerulein-treated mice during the observation period. Bombesin elicits lethal hemorrhagic-necrotizing pancreatitis in humanized PRSS1^R122H^-PRSS2 mice. (A) Gross pancreas and ascites 24 h after induction with high dose of cerulein or bombesin, illustrating distinct pancreatitis phenotypes (*n* = 9 per group). (B) Representative H&E images. In wild-type (WT) C57BL/6J mice, cerulein induces mild interstitial edema, whereas bombesin does not elicit pancreatitis. In heterozygous PRSS1^R122H^-PRSS2 mice, cerulein causes marked edematous pancreatitis, while bombesin produces severe hemorrhagic-necrotizing pancreatitis characterized by lobular necrosis and intralobular hemorrhage. (*n* = 9 per group). Scale bar, 200 μm. (C) Quantification of acinar necrosis after high-dose cerulein or bombesin. Mean ± s.d. (*n* = 6 per group). One-way ANOVA with Tukey’s test; *P* < 0.01 (**). (D) Representative CD31 (endothelial) and α-SMA (vascular smooth muscle) immunostaining demonstrating preserved vascular integrity after cerulein and disrupted vascular architecture following bombesin treatment (n = 5 per group). Scale bar, 200 μm. (E) Human cases of hemorrhagic-necrotizing pancreatitis show similar histopathology, including red blood cell extravasation and lobular ischemic coagulative necrosis.

To test whether distinct secretagogue stimuli drive divergent pancreatitis phenotypes, C57BL/6J mice and heterozygous PRSS1^R122H^-PRSS2 mice were treated with either cerulein or bombesin using eight hourly high-dose injections (100 μg/kg), and pancreatic injury was assessed 24 hours after the initial dose (Supplementary Fig. 1A). Cerulein produced mild edematous pancreatitis in C57BL/6J mice and a more pronounced edematous response with clear ascites in PRSS1^R122H^-PRSS2 mice (Fig. 1A). In contrast, bombesin caused no detectable edema in C57BL/6J mice but strikingly triggered a hemorrhagic necrotizing phenotype with large volumes of bloody ascites in PRSS1^R122H^-PRSS2 mice (Fig. 1A).

To contextualize these gross findings, pancreatic water content and serum amylase were quantified. Cerulein modestly increased both parameters in C57BL/6J mice, whereas bombesin had minimal effect. In PRSS1^R122H^-PRSS2 mice, cerulein and bombesin produced comparably elevated edema and serum amylase relative to wild-type (WT) controls (Supplementary Fig. 1B, C), indicating that conventional injury indices do not explain the profound phenotype divergence.

Histological analysis further separated these responses. Cerulein induced edema, inflammatory infiltration, and scattered acinar necrosis that were more pronounced in PRSS1^R122H^-PRSS2 mice than in C57BL/6J mice. In sharp contrast, bombesin provoked extensive intralobular hemorrhage, vascular disruption, and lobular ischemic necrosis exclusively in PRSS1^R122H^-PRSS2 mice while causing no detectable acinar injury in C57BL/6J mice (Fig. 1B). Morphometric quantification confirmed substantially greater lobular necrosis after bombesin than after cerulein in PRSS1^R122H^-PRSS2 mice (Fig. 1C).

Because vascular injury defined the bombesin phenotype, vascular integrity was examined directly. Bombesin treatment resulted in loss of CD31-positive endothelial continuity and disruption of α–smooth muscle actin–positive vascular smooth muscle layers, whereas vascular architecture remained intact after cerulein (Fig. 1D). To assess whether this phenotype recapitulates human disease, we examined human pancreatitis tissues obtained at autopsy. The histological features of bombesin-treated PRSS1^R122H^-PRSS2 pancreata exhibited with red blood cell extravasation, loss of vascular integrity, and lobular ischemic coagulative necrosis with minimal inflammatory infiltrates, closely resembled human hemorrhagic necrotizing pancreatitis (Fig. 1E). Areas of lobular necrosis without inflammatory infiltration indicated complete interruption of the local blood supply (Fig. 1E). In human severe acute pancreatitis, mortality rates can reach up to 40%^10^. Strikingly, no mortality was observed following high-dose cerulein treatment, whereas approximately 50% mortality occurred in bombesin-treated mice (Fig. 1F).

Additional histological features of bombesin-induced pancreatitis, assessed by focused hematoxylin and eosin (HE) staining, revealed intrapancreatic thrombosis and regions of lobular necrosis accompanied by intense inflammatory infiltration, consistent with impaired but incompletely abolished perfusion (Supplementary Fig. 2A, B). The same H&E analysis demonstrated structural damage to pancreatic islets following bombesin treatment (Supplementary Fig. 2C). Cleaved caspase-3 staining localized predominantly to the islet surface after bombesin, with minimal staining in acinar cells. In contrast, cerulein-induced pancreatitis exhibited the opposite pattern, with prominent acinar cleaved caspase-3 staining and relative sparing of islets (Supplementary Fig. 2D). Consistent with endocrine involvement, bombesin-treated mice showed a trend toward elevated fasting blood glucose and impaired intraperitoneal glucose tolerance (Supplementary Fig. 2E, F).

### Total intrapancreatic protease activity is uncoupled from pancreatitis severity.

Inappropriate intra-acinar trypsinogen activation is widely viewed as an initiating event in acute pancreatitis and is often assumed to scale with disease severity^21, 22^. Consistent with this paradigm, cerulein induced significantly higher total intrapancreatic protease activity, including trypsin and chymotrypsin, than bombesin in both C57BL/6J and heterozygous PRSS1^R122H^-PRSS2 mice (Fig. 2A, B). Paradoxically, despite lower total protease activity, bombesin produced markedly more severe injury in PRSS1^R122H^-PRSS2 mice, as defined by the hemorrhagic necrotizing phenotype as shown in Fig. 1.

To quantify this dissociation, pancreatitis severity was assessed by blinded semi-quantitative scoring of whole pancreatic sections, including hemorrhage, edema, acinar cell death, and inflammatory cell infiltration. These analyses showed that histologic severity did not track with total intrapancreatic protease activity (Fig. 2C–F). Notably, edema, acinar cell death, and inflammatory infiltration scores were comparable between cerulein- and bombesin-treated PRSS1^R122H^-PRSS2 mice despite substantial differences in total trypsin and chymotrypsin activity.

To further define inflammatory patterns associated with these divergent injury programs, immune cell infiltration was examined by CD11b and F4/80 staining. Cerulein-induced inflammation was concentrated in interlobular regions, whereas bombesin-induced pancreatitis showed pronounced regional heterogeneity, with areas of intense inflammatory infiltration interspersed with regions showing reduced or absent CD11b and F4/80 immunoreactivity (Supplementary Fig. 3). Loss of detectable extracellular epitopes on infiltrating immune cells is consistent with proteolytic cleavage by leaked digestive enzymes. Together, these findings indicate that severity and subtype cannot be inferred from total protease activity alone.

### Chronic sequelae of bombesin-induced hemorrhagic necrotizing pancreatitis in PRSS1 ^R122H^-PRSS2 mice.

Bombesin-induced pancreatitis severity increased in a dose-dependent manner in PRSS1^R122H^-PRSS2 mice, as demonstrated by escalating hemorrhage, acinar necrosis, edema, and ascites formation with increasing numbers of bombesin injections (Supplementary Fig. 4A-F). Having established this graded acute injury response, we next examined the long-term consequences of bombesin-induced hemorrhagic pancreatitis.

By day 21 after injury, bombesin-treated PRSS1^R122H^-PRSS2 mice developed striking chronic pancreatic abnormalities that were not observed following cerulein-induced pancreatitis. Gross examination revealed pancreatic cyst formation and amylase-rich ascites (Fig. 3A-C). Bombesin-treated mice also exhibited significant body weight loss and markedly reduced pancreatic mass (Fig. 3D, E), indicating extensive and largely irreversible pancreatic injury. Histological analysis confirmed cystic remodeling, widespread loss of acinar tissue, and disruption of pancreatic ductal architecture (Fig. 3F).

Notably, the combination of pancreatic duct disruption and enzyme-rich ascites closely resembles disconnected pancreatic duct syndrome, a recognized complication affecting approximately 30–50% of patients with necrotizing pancreatitis^23, 24^. These findings indicate that bombesin-induced hemorrhagic pancreatitis in PRSS1^R122H^-PRSS2 mice progresses to a chronic disease state that captures key structural and functional sequelae observed in severe human pancreatitis.

### Bombesin-induced hemorrhagic necrotizing pancreatitis is not PRSS1 ^R122H^ mutation-specific and is modulated by trypsin gene dosage.

The bombesin-induced hemorrhagic necrotizing pancreatitis phenotype was initially identified in PRSS1^R122H^-PRSS2 mice. To determine whether this phenotype depends on the PRSS1^R122H^ mutation, we examined a complementary transgenic mouse line expressing wild-type human PRSS1 and PRSS2 (PRSS1–PRSS2 mice). Bombesin administration using the same stimulation regimen induced pathological features indistinguishable from those observed in PRSS1^R122H^-PRSS2 mice, demonstrating that the hemorrhagic necrotizing phenotype is not dependent on the PRSS1^R122H^ mutation (Fig. 4A-H). The phenotype was stronger in homozygous mice than in heterozygous and correlated with trypsin expression and activity levels (Fig. 4A-H), suggesting trypsin activity still plays a critical role in the initiation of HNP.

### Enzyme accumulation within the pancreatic duct is a defining feature of hemorrhagic necrotizing pancreatitis.

**Figure 5:**
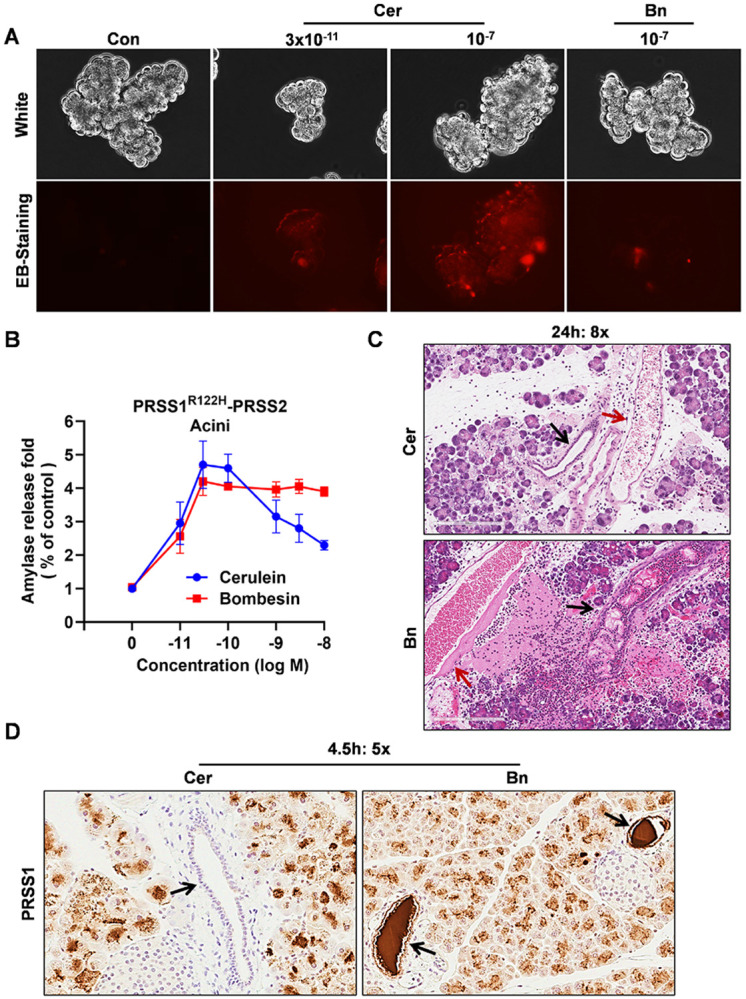
Pancreatic ductal enzymes accumulation in PRSS1^R122H^-PRSS2 (A) Primary pancreatic acinar cells from PRSS1^R122H^-PRSS2 mice were stimulated with saline (control), cerulein, or bombesin as indicated concentrations. Ethidium bromide (EB) was included to monitor membrane permeability. High-dose cerulein triggered membrane blebbing and ethidium bromide (EB; red) uptake, indicating loss of plasma-membrane integrity. In contrast, low-dose cerulein and bombesin caused no detectable cellular injury (Representative images from 3 independent experiments). (B) Schematic showing acinar responses: cerulein elicits biphasic amylase secretion curve, whereas bombesin sustains secretion even at high dose. Mean ± s.d. (representative from 3 independent experiments). (C) Representative histology showing ductal and vascular injury after high-dose bombesin, but not after high-dose cerulein administered using the same protocol (100 μg/kg, 8 × hourly injections) (n = 10 per group). Scale bars, 200 μm. (D) PRSS1 immunostaining shows marked intraductal enzyme accumulation and epithelial lining positivity after high-dose bombesin, but not cerulein. Representative images from five independent samples. Scale bars, 200 μm.

Given that bombesin induces lower total intrapancreatic trypsin activity than high-dose cerulein which produces only edematous pancreatitis, the development of severe hemorrhagic necrotizing pancreatitis following bombesin stimulation is paradoxical. To determine whether this difference reflects direct acinar cell injury, freshly isolated primary pancreatic acinar cells were exposed to cerulein or bombesin *in vitro*. High-dose cerulein induced overt acinar cell damage, characterized by membrane blebbing and ethidium bromide uptake, whereas bombesin and low-dose of cerulein did not cause detectable acinar cell injury (Fig. 5A). These findings indicate that bombesin-induced pancreatitis is not initiated by direct acinar cell cytotoxicity of bombesin receptor activation.

The enzyme secretion of pancreatic acinar cells in response secretagogues was in a stimulus- and concentration-dependent manner^25, 26, 27^. Low concentrations of cholecystokinin (CCK) or its analog cerulein stimulate enzyme secretion, whereas supraphysiologic concentrations suppress secretion despite continued intracellular enzyme activation. In contrast, bombesin activates distinct intracellular signaling pathways and preserves the secretion-associated cytoskeletal architecture, thereby failing to induce high-dose secretory inhibition (Fig. 5B). Based on these differences, we hypothesized that bombesin-induced enzyme activation occurs in the setting of preserved secretion, resulting in the delivery of activated digestive enzymes into the pancreatic ductal compartment.

Consistent with this hypothesis, histological analysis revealed prominent ductal dilation, disruption, and obstruction accompanied by adjacent vascular injury in bombesin-treated mice, whereas ductal architecture remained largely intact following high-dose cerulein stimulation (Fig. 5C). Notably, this combined pattern of ductal and vascular injury closely mirrors that observed in human hemorrhagic necrotizing pancreatitis^28^, as illustrated in representative human pancreatic pathology images curated by the Japanese Society of Pathology (https://pathology.or.jp/corepicturesEN/11/c04/04.html). To directly assess ductal enzyme accumulation, immunohistochemical staining for PRSS1 was performed at the examined time point. No detectable PRSS1 immunoreactivity was observed in ducts from cerulein treated mice, whereas ducts from bombesin treated mice contained abundant PRSS1 positive material within the ductal lumen and along the ductal epithelial lining (Fig. 5D), supporting intraductal accumulation of trypsinogen and its association with ductal injury. Together, these findings identify accumulation of activated digestive enzymes within the ductal compartment, as a key mechanistic feature distinguishing hemorrhagic necrotizing pancreatitis from edematous disease.

### Low-dose cerulein paradoxically triggers severe hemorrhagic necrotizing pancreatitis through a duct-centered mechanism, whereas high-dose cerulein causes edematous disease.

Based on the dose-dependent effects of cerulein on secretion (Fig. 5B), we reasoned that cerulein administered at doses that preserve enzyme secretion would recapitulate the bombesin-induced hemorrhagic pancreatitis phenotype. To test this hypothesis, PRSS1^R122H^-PRSS2 mice received a single intraperitoneal injection of cerulein across a wide dose range from 2.5 to 100 μg/kg (Supplementary Fig. 5A). Indeed, cerulein induced a dose dependent hemorrhagic phenotype, with maximal hemorrhage observed at doses of 10 to 15 μg/kg, and paradoxically hemorrhage diminished with higher doses of cerulein at 50 to 100 μg/kg (Fig. 6A). At hemorrhage inducing doses (10 to 15 μg/kg), pancreatic histology closely resembled that observed following bombesin stimulation and was characterized by prominent ductal dilation, ductal disruption, and adjacent vascular injury (Fig. 6B). Notably, under these conditions, the severity of hemorrhagic injury was uncoupled from total intrapancreatic trypsin activity, which did not scale with hemorrhage severity across doses (Fig. 6C and Supplementary Fig. 5D, E). Pancreatic edema and serum amylase levels increased with doses and plateaued above approximately 20 μg/kg, suggesting that these parameters do not necessarily correlate with pancreatitis severity (Supplementary Fig. 5B and C). At the lowest cerulein dose tested of 2.5 μg/kg, mice developed only mild edematous pancreatitis without hemorrhage (Fig. 6A, B), indicating that a threshold level of secretagogue induced trypsin activity is required to initiate hemorrhagic necrotizing injury. This threshold requirement likely explains why bombesin fails to induce pancreatitis in wild type mice but provokes severe hemorrhagic disease in transgenic mice expressing human PRSS1 and PRSS2.

Time course analysis further supported a duct centered mechanism. By day 7 following lower dose cerulein stimulation at hemorrhage inducing doses, pancreatic ducts were dilated and filled with debris, closely resembling the changes observed in bombesin treated mice (Fig. 6D, right panel). In contrast, mice receiving high dose cerulein showed no evidence of intraductal debris at the same time point (Fig. 6D, left panel). By day 21, abundant intraductal protein plugs with amorphous calcification had formed in lower dose cerulein treated mice, closely mimicking features of severe pancreatitis in human (Fig. 6E). Together, these findings show that the development of hemorrhagic necrotizing pancreatitis requires both sufficient trypsin activity and preserved enzyme secretion that leads to digestive enzyme accumulation within the pancreatic ductal compartment, and that hemorrhagic severity is not determined by total intrapancreatic protease activity alone.

### Pharmacologic flush-out of ductal enzyme accumulation prevents hemorrhagic necrotizing pancreatitis.

Guided by the duct centered mechanism identified above, we postulated that reducing intraductal digestive enzyme burden could prevent progression to hemorrhagic necrotizing pancreatitis. To model a therapeutic intervention, treatment was initiated 2.5 h after bombesin challenge, a time point at which ductal alterations and early pancreatic injury are detectable (Supplementary Fig. 6A). Secretin is a peptide hormone that stimulates pancreatic ductal epithelial cells to secrete bicarbonate rich fluid and is routinely used in pancreatic function testing and enhanced magnetic resonance cholangiopancreatography^29, 30^. We hypothesized that secretin induced ductal fluid secretion might mitigate intraductal enzyme accumulation. However, when administered alone at 2.5 h after bombesin challenge, secretin did not confer protection and instead modestly exacerbated hemorrhagic injury, likely reflecting increased intraductal pressure in the setting of impaired pancreatic outflow (Fig. 7A-G). We next reasoned that effective ductal flush-out would require not only enhanced fluid secretion but also relaxation of the sphincter of Oddi, the muscular valve regulating pancreatic outflow into the duodenum. Accordingly, we administered the nitric oxide donor isosorbide mononitrate (ISMN), which relaxes the sphincter of Oddi^31, 32, 33, 34, 35, 36^. Although ISMN alone conferred no protection, its combination with secretin completely prevented the development of hemorrhagic pancreatitis (Fig. 7B-G). Collectively, these findings support the duct-centered mechanism and identify a potentially translatable therapeutic strategy, as both agents are FDA approved and amenable to rapid clinical evaluation. A schematic overview of the proposed mechanism and therapeutic concept is provided (Graphical abstract).

## DISCUSSION

In this study, we identify intraductal digestive enzyme accumulation as a key determinant of severe hemorrhagic necrotizing pancreatitis. Retention of active proteases within the pancreatic ductal system leads to ductal disruption, parenchymal necrosis, and hemorrhagic injury. This duct-initiated mechanism explains pathological features of fulminant pancreatitis that are inadequately captured by existing experimental models and reframes disease severity from one driven primarily by intracellular acinar activation to one in which failure of ductal clearance plays a decisive role. These findings further suggest that reducing intraductal enzyme burden through pharmacologic ductal flush-out can attenuate disease progression, highlighting the pancreatic duct as a previously underappreciated translational target.

Most experimental models of acute pancreatitis emphasize acinar cell–centric mechanisms, particularly inappropriate intracellular digestive enzyme activation^8, 9^. Hyperstimulation models such as cerulein-induced pancreatitis produce robust acinar stress, inflammation, and edema and can induce necrosis at sufficient intensity, yet injury remains largely driven by intracellular acinar responses^37^. Likewise, the choline-deficient, ethionine-supplemented diet model induces severe pancreatic injury through systemic metabolic stress and acinar toxicity without addressing ductal clearance failure^38, 39^. In contrast, bile acid–based or retrograde infusion models rely on nonphysiologic chemical injury and artificial delivery routes that obscure endogenous disease processes^40, 41, 42^. The duct-driven hemorrhagic pancreatitis described here differs fundamentally by arising from failure of physiological enzyme clearance, resulting in intraductal protease accumulation, ductal disruption, and secondary vascular injury, a distinction that likely explains why existing models incompletely reproduce the hemorrhagic phenotype seen in severe human disease.

These findings indicate that the pancreatic duct is not merely a passive conduit for exocrine output but can determine disease trajectory when luminal clearance fails^43, 44, 45^. Mechanistically, our data support a two-requirement model for hemorrhagic necrotizing pancreatitis: a threshold level of protease activity is required to initiate catastrophic injury, and secretion must remain sufficiently intact to deliver enzymes into the ductal system, enabling intraductal accumulation. This framework reconciles divergent phenotypes across stimulation paradigms. Supraphysiologic cerulein suppresses secretion despite robust intracellular activation, limiting ductal enzyme delivery^26, 46, 47, 48^, whereas bombesin and moderate-dose cerulein preserve secretion and favor ductal exposure to activated or activatable proteases^27, 49, 50^. Once proteases accumulate intraductally, enzymatic activity becomes spatially concentrated within a confined compartment, promoting focal ductal disruption and enzyme escape into the interstitium. Protease dissemination near the periductal microvasculature provides a mechanistic link to vascular barrier failure and hemorrhage. Accordingly, bulk pancreatic protease activity is an incomplete surrogate for disease severity because it does not capture where enzymatic activity is exerted.

These mechanistic insights identify ductal washout as a tractable therapeutic axis while indicating that enhancing secretion alone is insufficient when pancreatic outflow is impaired. In a clinically relevant intervention paradigm initiated 2.5 hours after bombesin challenge, when early ductal alterations are detectable but before overt hemorrhage, secretin failed to confer protection and modestly exacerbated hemorrhagic injury, likely by increasing intraductal pressure in the setting of impaired drainage^51, 52, 53^. This observation implies that effective enzyme washout requires both enhanced secretion and restoration of downstream outflow, specifically relaxation of the sphincter of Oddi^36, 54, 55, 56^. Although the nitric oxide donor isosorbide mononitrate was ineffective as monotherapy, its combination with secretin completely prevented hemorrhagic pancreatitis. Together, these findings support a mechanism-based strategy in which coordinated enhancement of secretion and ductal drainage reduces intraductal protease burden, preserves ductal integrity, and prevents secondary vascular injury. Because both agents are FDA approved for established clinical indications, near-term translational evaluation of this approach is feasible.

The chronic sequelae following duct-driven hemorrhagic injury extend the implications of intraductal protease retention beyond the acute phase. By day 21 after bombesin challenge, mice exhibited profound pancreatic remodeling characterized by loss of acinar tissue, reduced pancreatic mass, cyst formation, and amylase-rich ascites, indicating persistent leakage of enzyme-containing fluid from a disrupted ductal system. Sustained architectural distortion of the ductal compartment and intraductal protein plugs with amorphous calcification were observed, consistent with ongoing stasis and obstruction. This constellation closely resembles disconnected pancreatic duct syndrome^57, 58, 59^, a frequent and therapeutically challenging complication of necrotizing pancreatitis, suggesting that early ductal injury can initiate a self-reinforcing cycle of impaired drainage, continued enzyme leakage, and progressive remodeling.

In summary, our study establishes intraductal digestive enzyme accumulation and impaired ductal clearance as defining mechanisms that drive hemorrhagic necrotizing pancreatitis. By distinguishing duct-driven hemorrhagic injury from acinar-centric pancreatitis programs, these findings explain why bulk pancreatic protease activity does not reliably predict severity and identify the ductal compartment as a decisive site of pathogenic enzyme action. The ability to prevent catastrophic hemorrhagic disease by co-optimizing ductal secretion and pancreatic outflow supports a translatable therapeutic principle centered on restoring ductal clearance and motivates future efforts to develop clinical measures of ductal patency and drainage to enable timely intervention in this high-risk pancreatitis subtype.

## Supplementary Material

This is a list of supplementary files associated with this preprint. Click to download.

- Supplementarymaterialsandmethods.docx
- SupplementalTable1.docx
